# Transcription Factor TCP9 Enhances *Arabidopsis* Tolerance to Cadmium Toxicity via *ZAT6* and *ZAT10* Activation

**DOI:** 10.3390/plants15172563

**Published:** 2026-08-24

**Authors:** Jianju She, Feng Chen, Jiayi Liu, Yan He, Jiafei Xie, Lu Li, Ting Li, Jinhui Lin, Fengfeng Dang

**Affiliations:** 1 Shaanxi Key Laboratory of Research and Utilization of Resource Plants on the Loess Plateau, College of Life Sciences, Yan’an University, Yan’an 716000, China; sjj@yau.edu.cn (J.S.); chenfengyadx@163.com (F.C.); ljy55635@163.com (J.L.); hya@163.com (Y.H.); jfxie2026@163.com (J.X.); lilu0611@163.com (L.L.); tingli4317@163.com (T.L.); 2 Fruit Research Institute, Fujian Academy of Agricultural Sciences, Fuzhou 350003, China

**Keywords:** cadmium toxicity, TCP9, ZAT6, ZAT10

## Abstract

Cadmium (Cd), a highly toxic and mobile heavy metal, has emerged as a severe environmental concern in global agroecosystems, posing a substantial threat to human health. Although prior studies have established that *ZAT6* and *ZAT10* positively regulate *Arabidopsis* tolerance to Cd toxicity, the underlying molecular mechanisms remain largely elusive. The present study provides evidence that a class I TCP transcription factor, TCP9, significantly enhances *Arabidopsis* tolerance to Cd toxicity through the direct activation of *ZAT6* and *ZAT10* expression. The real-time quantitative PCR (RT-qPCR) analysis indicates that the expression of *TCP9* was induced under Cd toxicity. Meanwhile, the *tcp9* mutant exhibited heightened sensitivity to Cd toxicity, accompanied by elevated Cd accumulation in both shoots and roots. Notably, the complemented lines exhibited phenotypic characteristics analogous to those observed in the wild-type (WT) plants. Further physiological and biochemical analyses revealed that, in comparison to WT, the *tcp9* mutant displayed elevated hydrogen peroxide (H_2_O_2_) accumulation and reduced contents of catalase (CAT), ascorbate peroxidase (APX), and peroxidase (POD) under Cd toxicity. Furthermore, TCP9 directly interacted with the promoters of *ZAT6* and *ZAT10* in vitro, facilitating their transcription and consequently enhancing plant tolerance to Cd toxicity. Overall, our findings showed that TCP9 enhances Cd tolerance via modulating *ZAT6* and *ZAT10*, thereby identifying TCP9 as a potential key target for improving plant tolerance to Cd toxicity.

## 1. Introduction

Cadmium (Cd), recognized as one of the most toxic heavy metal pollutants, is accumulating in soil at an increasingly alarming rate [1]. This element is readily absorbed and translocated by plant roots, and its accumulation in plant tissues leads to biomagnification along the food chain, thereby posing a significant threat to human health [2,3]. Within plant cells, Cd induces a surge in reactive oxygen species (ROS), including singlet oxygen (^1^O_2_), superoxide radical (O_2_^−^), hydrogen peroxide (H_2_O_2_), and hydroxyl radical (·OH). Among these, H_2_O_2_ is particularly detrimental due to its relatively long half-life, leading to cellular oxidative damage and programmed cell death [4,5,6]. Furthermore, Cd disrupts the homeostasis of essential metal ions in plants, which severely hinders their normal growth and development [7,8]. As a consequence of post-industrial civilization, Cd acts as a stressor for which plants have not evolved specific tolerance mechanisms. Nevertheless, plants can mitigate Cd toxicity through various strategies, including controlling Cd influx, chelating Cd, enhancing Cd efflux, sequestering and remobilizing Cd, and scavenging ROS induced by Cd exposure.

As a divalent cation, Cd enters plant cells largely by hijacking transporters intended for the uptake of essential metal ions, such as iron (Fe) and zinc (Zn). These transporters include iron-regulated transporters (IRTs), zinc-regulated transporters (ZRTs), natural resistance-associated macrophage proteins (NRAMPs), and heavy metal ATPases (HMAs), as specific transporters or channels dedicated to Cd uptake have not yet been identified [9,10,11]. During root uptake, Cd competes with Fe and Zn, thereby exacerbating their deficiency. This competitive interaction not only induces symptoms akin to Fe deficiency, such as leaf chlorosis, but also impairs the essential function of Fe and Zn in the synthesis and activation of the antioxidant system [5,7]. Plants mitigate Cd toxicity through two principal strategies, one of which is the use of metal transport proteins to regulate Cd uptake and facilitate vacuolar sequestration [12,13]. Secondly, they depend on small molecules, such as glutathione (GSH) and phytochelatins (PC), to effectively chelate and detoxify Cd [14,15]. Simultaneously, the antioxidant enzyme systems, comprising SOD, CAT, POD, glutathione reductase (GR), and ascorbate peroxidase (APX), are activated in a synergistic manner. This activation plays a critical role in scavenging excessive ROS induced by Cd exposure and in maintaining cellular redox homeostasis [5,16,17,18,19]. Although substantial progress has been achieved in deciphering plant responses to Cd toxicity, the underlying molecular mechanisms are not yet fully elucidated.

Emerging evidence underscores the pivotal regulatory role of transcription factors (TFs) in mediating plant responses to Cd stress [20,21]. These factors bind to specific *cis*-acting elements within the promoters of target genes, thereby orchestrating transcriptional activation or repression of a suite of functional genes. This regulatory mechanism facilitates a swift and precise response to Cd stress. To date, numerous TF families have been identified as integral to the regulation of plant Cd detoxification. For instance, bHLH38 and bHLH39, members of the basic helix-loop-helix (bHLH) family, indirectly modulate Cd uptake by regulating *IRT1* and *FRO2*, which are critical genes in the Fe uptake pathway [9,22]. Similarly, MYB4, a member of the MYB family, influences cell wall composition by regulating genes associated with lignin synthesis, thus affecting Cd immobilization and accumulation [23]. Notably, MYB49 directly binds to the promoter regions of *bHLH38* and *bHLH101*, thereby modulating Cd uptake in plants by regulating *IRT1* expression. Moreover, MYB49 interacts with ABI5 through the abscisic acid (ABA) signaling pathway to mitigate Cd accumulation in plants [24]. WRKY TFs, including WRKY12 and WRKY13, are pivotal in Cd detoxification by regulating the expression of genes associated with GSH metabolism [25,26]. A positive feedback mechanism exists between *CaWRKY41* and H_2_O_2_, which increases the sensitivity of pepper plants to Cd stress [5]. Similarly, *ZAT10* enhances *Arabidopsis* tolerance to Cd stress by suppressing Cd-induced H_2_O_2_ accumulation and *IRT1* expression [16]. In addition, Cd stress triggers the expression of *ZAT6*, which subsequently enhances the Cd tolerance of *Arabidopsis* by upregulating *GSH1* and genes involved in PC biosynthesis [14]. Nonetheless, the current understanding of the regulatory network governing the Cd stress response is incomplete. Moreover, the key regulatory factors that integrate upstream signals and orchestrate multiple downstream defensive pathways remain unidentified. Additionally, existing research has primarily concentrated on the regulation of individual downstream target genes by specific TFs. The molecular mechanisms through which TFs systematically confer Cd tolerance via the coordinated regulation of multiple downstream TFs are not yet well elucidated.

TCP TFs constitute a plant-exclusive family of transcriptional regulators, which are grouped into Class I (PCF-type) and Class II (CYC/TB1-type) owing to distinguishable features of their DNA-binding domains [27,28]. These factors are significantly involved in a range of growth and developmental processes, such as cell proliferation, leaf morphogenesis, and hormone signaling [29,30,31]. Recent evidence increasingly indicates that TCPs are pivotal in mediating plant responses to biotic and abiotic stresses [32,33]. For example, TCP8 and TCP14 are associated with pathogen defense [34,35,36], whereas TCP20 influences nitrogen starvation responses by regulating nitrate transporter genes [37]. In wheat, the TaTCP6-TaSPX-TaPHR2 module helps manage nitrogen and phosphorus use by triggering the phosphorus starvation response genes, thus decreasing the need for agricultural fertilizers [38]. Despite these advancements, the specific functions of TCPs in Cd toxicity remain largely unexplored. Beyond documented expression changes in certain TCP members following Cd exposure, their precise roles, regulatory networks, and molecular pathways have yet to be fully understood.

Previously, several other studies have revealed that TCP9 interacts with TCP20 to modulate jasmonic acid (JA) synthesis via direct binding to the JA biosynthesis gene *LOX2,* thereby regulating endogenous JA levels and influencing leaf development [30]. Meanwhile, TCP8 and TCP9 regulate the transcription of *ISOCHORISMATE SYNTHASE 1* (*ICS1*), a key gene in salicylic acid (SA) production, boosting SA levels and strengthening plant immunity against pathogens [34]. Additionally, TCP9 alters *Arabidopsis* root growth by managing ROS homeostasis, aiding in defense against nematode infection [39]. Here, we elucidated the novel functional role and regulatory mechanism of TCP9 in the Cd toxicity response. The expression of *TCP9* was markedly upregulated by Cd exposure. Through genetic analysis involving loss-of-function mutants and complementary transgenic lines, we verified that *TCP9* acts as a positive regulator of Cd tolerance in *Arabidopsis*. Mechanistically, TCP9 was demonstrated to directly bind to the promoters of *ZAT6* and *ZAT10*, thereby activating their transcription. Considering the documented roles of *ZAT6* and *ZAT10* in antioxidant defense and Cd detoxification, our findings reveal a mechanism by which *TCP9* systematically enhances Cd tolerance via the synergistic activation of downstream pleiotropic stress-responsive genes.

## 2. Results

### 2.1. Cd Stress Triggers TCP9 Expression, While the tcp9-3 Mutant Increases Cd Sensitivity in Arabidopsis

To elucidate the transcriptional regulatory mechanism underlying plant responses to Cd toxicity, we initially utilized approximately 200 *T-DNA* insertion mutant lines derived from members of the WRKY, ERF, bZIP, and TCP families as candidate lines for our study. Subsequently, using the wild type (WT) as a control, we performed a comprehensive phenotypic analysis of these *T-DNA* insertion mutants on 1/2 Murashige and Skoog (MS) medium supplemented with 0, 30, and 60 μM CdSO_4_. Our findings revealed that the SALK *T-DNA* insertion mutant, designated SALK_035853, exhibited a significantly reduced primary root length compared to the WT when cultivated on 1/2 MS medium supplemented with 30, 60, and 90 μM CdSO_4_ (Figure S1A–C). In contrast, no discernible growth differences were observed between the mutant and the WT under 1/2 MS media without Cd. A previous study has established that the *T-DNA* insertion site in this mutant (SALK_035853, referred to as *tcp9-3*) is located within the 5′ untranslated region (UTR) of *TCP9* [34].

**Figure 1:**
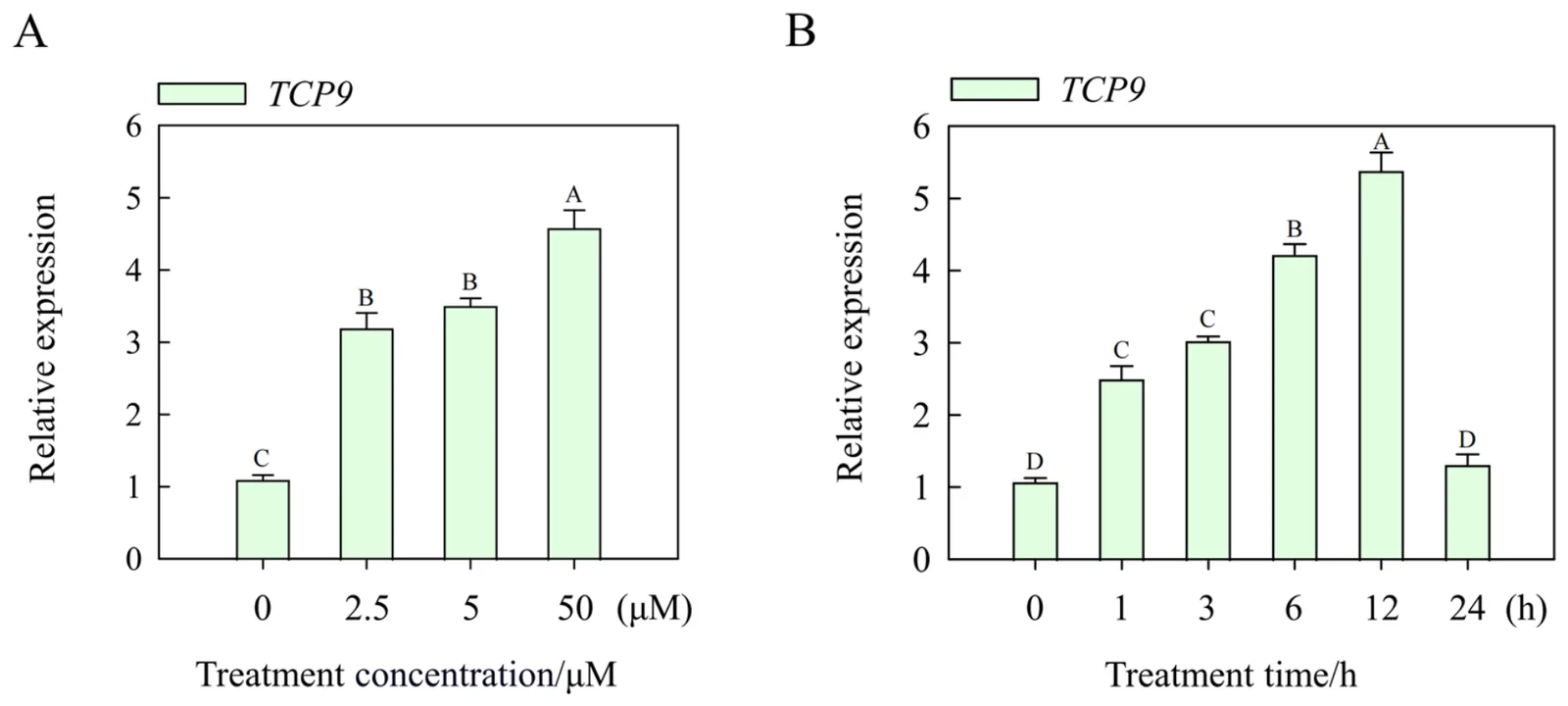
Cd toxicity greatly increased *TCP9* gene transcript. (**A**,**B**) Total RNA was extracted from 7-day-old WT seedlings exposed to CdSO_4_ at varying concentrations (0, 2.5, 5, and 50 μM) for 6 h (**A**) and at 30 μM for different durations (0, 1, 3, 6, 12, and 24 h) (**B**). Data represent the mean ± SE from three biological replicates. Different letters denote significant differences, as determined by Duncan’s multiple range test (*p* < 0.01).

Then, we conducted PCR amplification followed by DNA sequencing to verify the *T-DNA* insertion site in the *tcp9-3* mutant. The findings revealed that the *T-DNA* was inserted 83 bp (base pairs, bp) upstream of the ATG start codon of *TCP9* (AT2G45680), as illustrated in Figure S2. Based on these findings, we hypothesized that *TCP9* could play a role in modulating Cd tolerance in *Arabidopsis*. This hypothesis was further validated by detecting the transcriptional alteration of *TCP9* upon Cd exposure through RT-qPCR quantification. The data indicated that exposure to varying CdSO_4_ concentrations significantly elevated *TCP9* transcript levels (Figure 1A). Additionally, the temporal expression profile of *TCP9* under Cd stress was investigated. As depicted in Figure 1B, *TCP9* transcript levels progressively increased with treatment duration, peaking at 12 h post-treatment, before gradually returning to baseline levels. Collectively, *TCP9* is implicated in modulating *Arabidopsis* responses to Cd toxicity.

### 2.2. TCP9 Enhances Cd Tolerance in Arabidopsis

**Figure 2:**
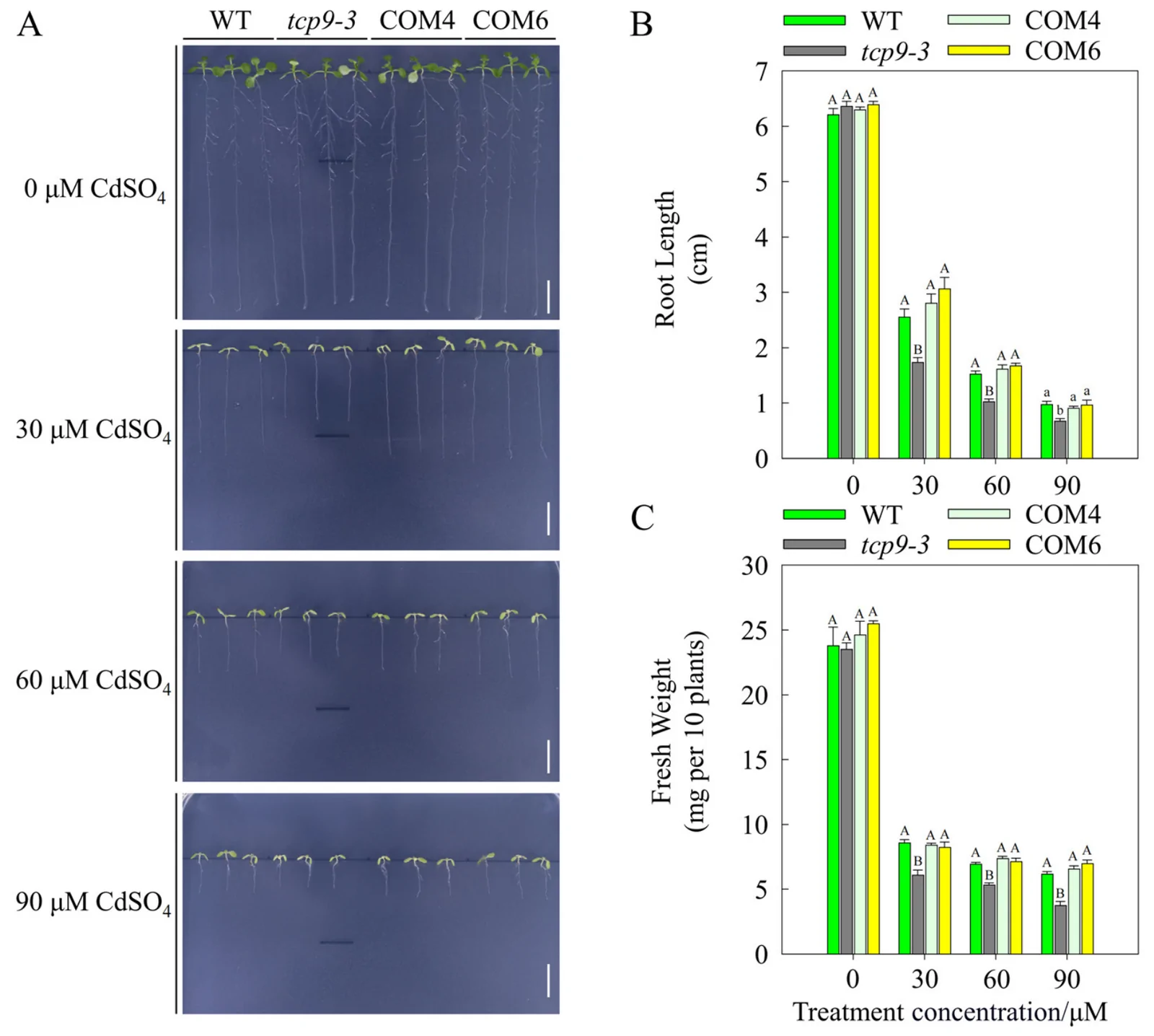
The function of *TCP9* in the response of *Arabidopsis* to Cd toxicity. (**A**) Phenotypic analysis was conducted on WT, *tcp9-3* mutant, and *TCP9pro:TCP9/tcp9-3* complementary lines (COM4 and COM6) under Cd toxicity. Seven-day-old seedlings were grown on 1/2 MS medium with or without 30, 60, and 90 μM CdSO_4_. Scale bar = 1 cm. Results were consistent across three independent experiments. (**B**,**C**) Root length (**B**) and fresh weight (**C**) of 7-day-old WT, *tcp9-3*, COM4, and COM6 seedlings grown vertically on a 1/2 MS agar plate with varying CdSO_4_ concentrations. Data represent the mean ± SE from three biological replicates. Statistical significance among the groups was assessed using Duncan’s multiple range test, where different letters were used to denote significant differences, with lowercase letters indicating a significance level of *p* < 0.05 and uppercase letters indicating a significance level of *p* < 0.01.

**Figure 3:**
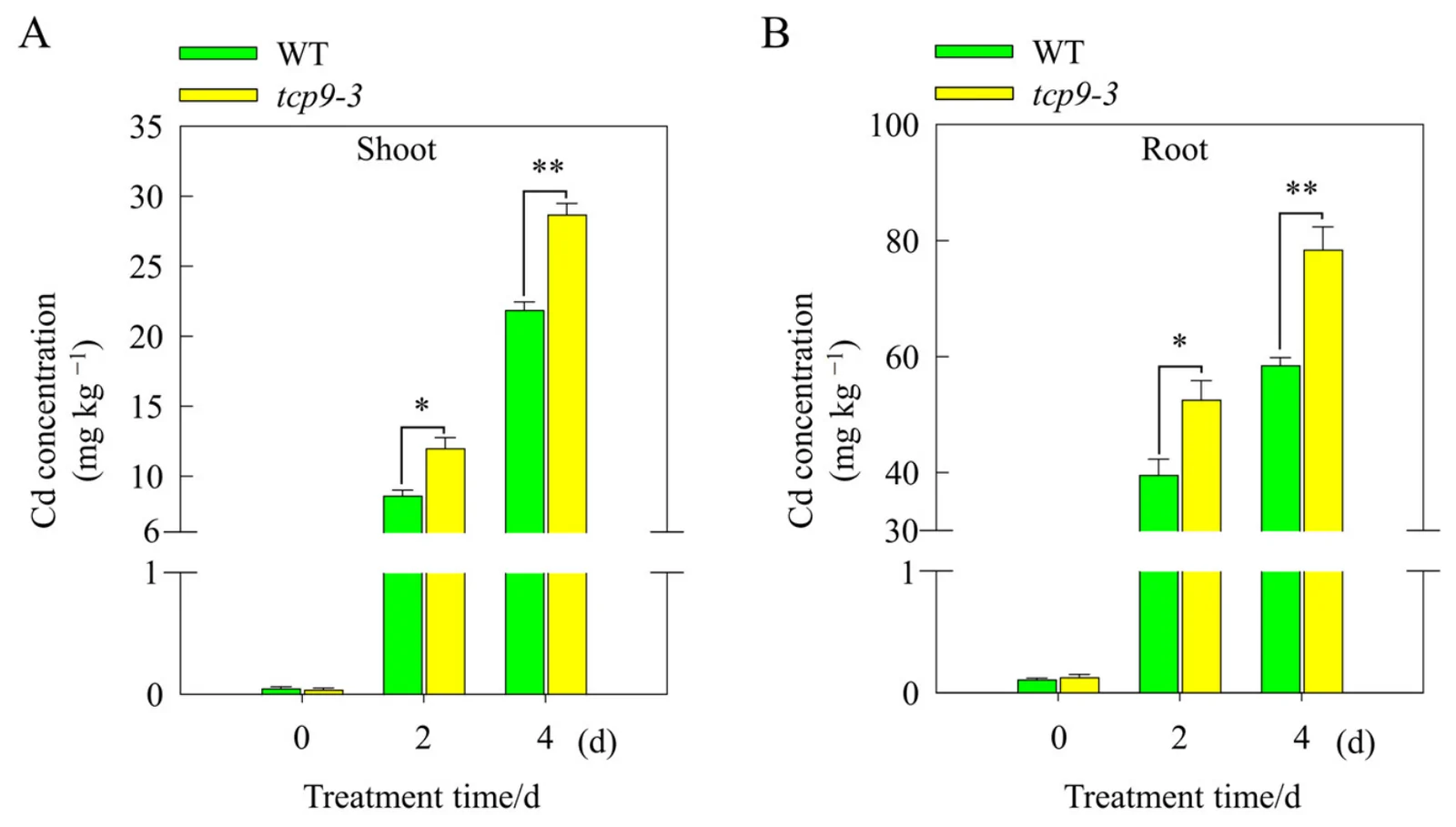
Cd accumulation changes in *Arabidopsis* WT and *tcp9-3* mutant seedlings under Cd toxicity. (**A**,**B**) Cd concentrations in the shoots (**A**) and roots (**B**) of WT and *tcp9-3* mutant plants under Cd toxicity were measured. Seven-day-old seedlings initially grown on 1/2 MS medium were moved to 1/2 MS medium with or without 30 μM CdSO_4_ for 0, 2, and 4 d. Data are presented as mean ± SE from three biological replicates, with asterisks indicating significant differences (Student’s *t*-test, * *p* < 0.05 or ** *p* < 0.01).

To elucidate the role of *TCP9* in Cd tolerance in *Arabidopsis*, we generated *TCP9pro*:*TCP9* complementation lines within the *tcp9-3* mutant background and selected two independent lines designated COM4 and COM6 (Figure S3) for further analysis. Subsequently, we assessed the growth of WT, *tcp9-3* mutant, and the complementation lines COM4 and COM6 on 1/2 MS medium supplemented with 0, 30, 60, and 90 μM CdSO_4_. The findings indicated that, in the absence of Cd, all genotypes exhibited comparable root lengths and fresh weights. However, Cd stress markedly impeded primary root elongation and overall growth in all plants. Compared with the WT, the *tcp9-3* mutant displayed enhanced sensitivity to Cd stress. Importantly, the two complementation lines, COM4 and COM6, showed root lengths and fresh weights comparable to those of the WT under Cd stress, thereby effectively complementing the Cd-sensitive phenotype of the *tcp9-3* mutant (Figure 2A–C). Additionally, WT and *tcp9-3* mutant plants were initially cultivated on 1/2 MS medium for 7 d before being transferred to 1/2 MS medium supplemented with 30 μM CdSO_4_. The Cd content in both shoots and roots was subsequently quantified on days 0, 2, and 4 following treatments. As illustrated in Figure 3A,B, the *tcp9-3* mutant exhibited significantly higher Cd accumulation in both shoots and roots compared to the WT. Collectively, these findings indicate that *TCP9* functions as a positive regulator of Cd tolerance in *Arabidopsis*.

### 2.3. TCP9 Mutation Leads to Elevated H_2_O_2_ Accumulation Under Cd Stress

**Figure 4:**
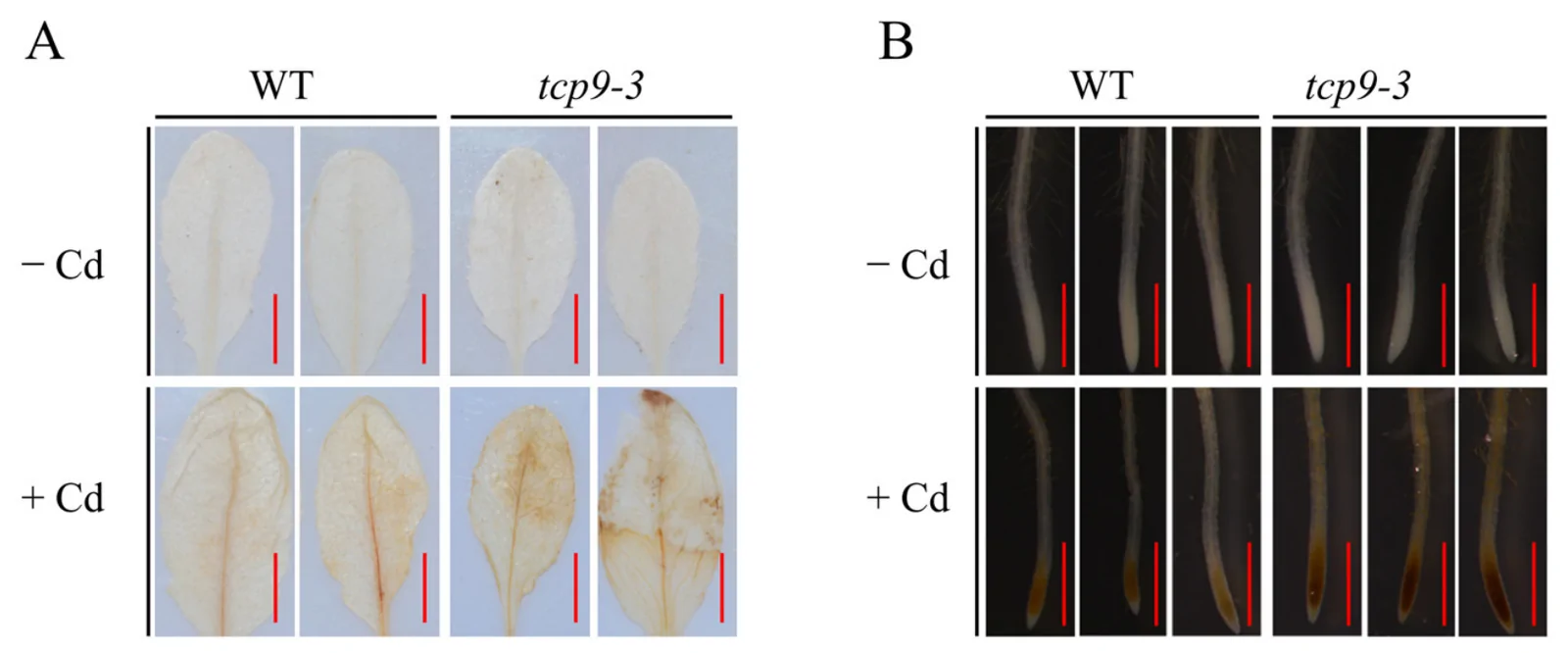
ROS levels differ in *Arabidopsis* WT and *tcp9-3* mutant seedlings under Cd toxicity. (**A**,**B**) DAB staining revealed H_2_O_2_ accumulation in the leaves (**A**) and roots (**B**) of WT and *tcp9-3*. Seedlings were exposed to 30 μM CdSO_4_ for 24 h. Scale bar = 1 cm.

**Figure 5:**
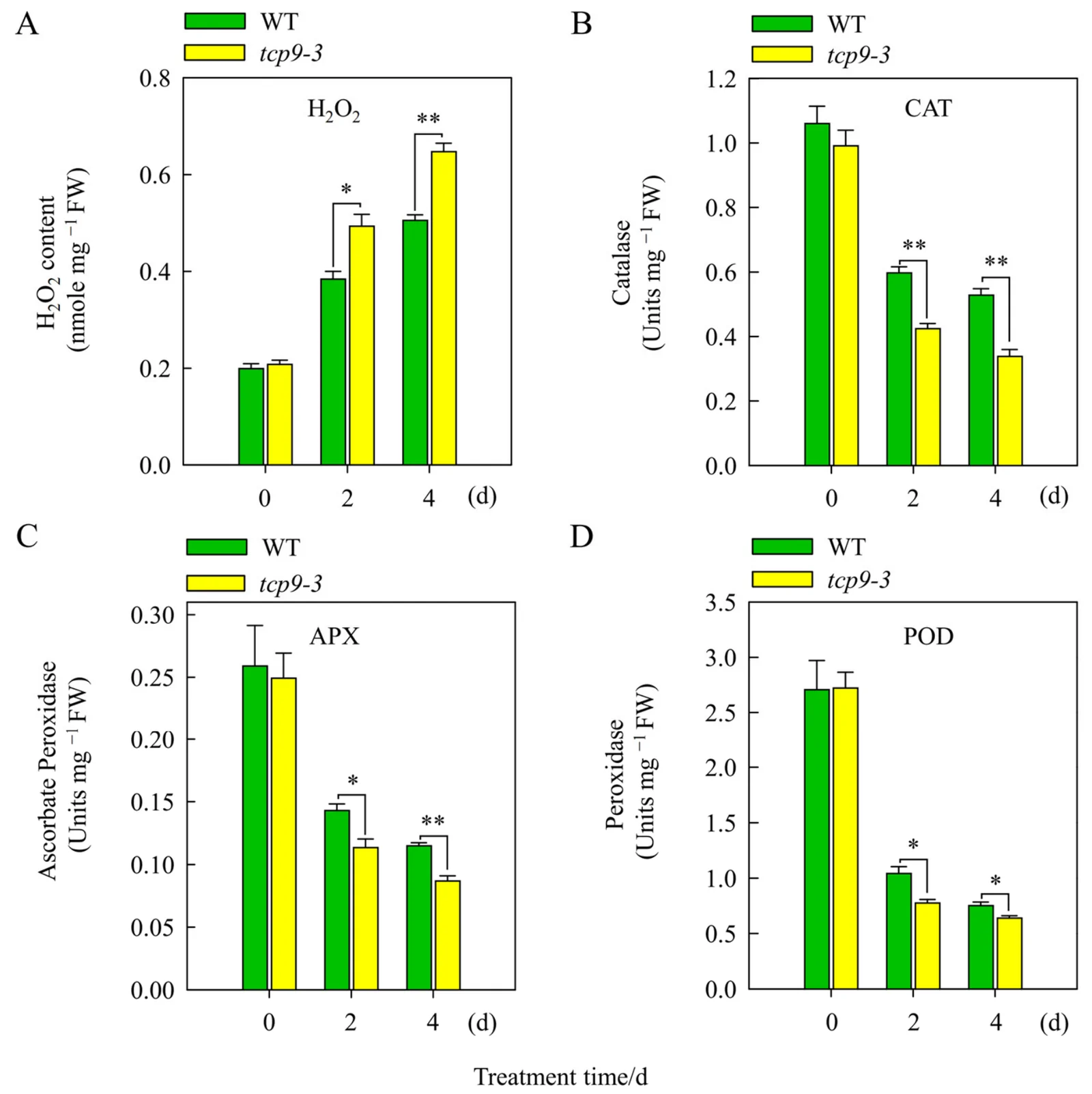
The *tcp9-3* seedlings showed increased H_2_O_2_ levels and reduced antioxidant enzyme contents under Cd toxicity. (**A**) H_2_O_2_ levels in the WT and *tcp9-3* mutant seedlings under Cd toxicity. (**B**–**D**) The contents of CAT (**B**), APX (**C**), and POD (**D**) were measured in WT and *tcp9-3* mutant seedlings exposed to Cd toxicity. Seven-day-old seedlings were transferred from 1/2 MS medium to the same medium with or without 30 μM CdSO_4_ for 0, 2, and 4 d, then harvested for analysis. Results are presented as mean ± SE from three biological replicates, with asterisks indicating significant differences (Student’s *t*-test, * *p* < 0.05 or ** *p* < 0.01).

One prevalent manifestation of Cd stress in plants is the induction of oxidative stress, which is characterized by the excessive accumulation of ROS [18]. Herein, we evaluated H_2_O_2_ accumulation in WT and tcp9-3 mutant plants under Cd stress via 3,3′-diaminobenzidine (DAB) histochemical staining. The staining assay demonstrated that H_2_O_2_ contents were markedly higher in the *tcp9-3* mutant relative to WT plants upon Cd exposure. Conversely, under normal conditions without Cd stress, no significant differences were observed in H_2_O_2_ accumulation between the *tcp9-3* mutant and the WT (Figure 4A,B). In addition, we conducted an analysis of the contents of H_2_O_2_ and antioxidant defense enzymes, specifically CAT, APX, and POD, in both the *tcp9-3* mutant and the WT plants on days 0, 2, and 4 following Cd exposure. The findings revealed that the H_2_O_2_ content in the *tcp9-3* mutant was significantly elevated compared to the WT on days 2 and 4 after Cd exposure (Figure 5A). Conversely, the contents of the antioxidant defense enzymes were markedly reduced in the *tcp9-3* mutant relative to the WT (Figure 5B–D). Under normal conditions without Cd exposure, no significant differences in H_2_O_2_ and antioxidant enzyme contents were detected between the *tcp9-3* mutant and the WT (Figure 5). Collectively, Cd exposure triggers more pronounced H_2_O_2_ accumulation in the *tcp9-3* mutant, indicating that TCP9 may boost plant tolerance by mitigating Cd-induced oxidative damage.

### 2.4. TCP9 Directly Promotes the Transcription of ZAT6 and ZAT10

**Figure 6:**
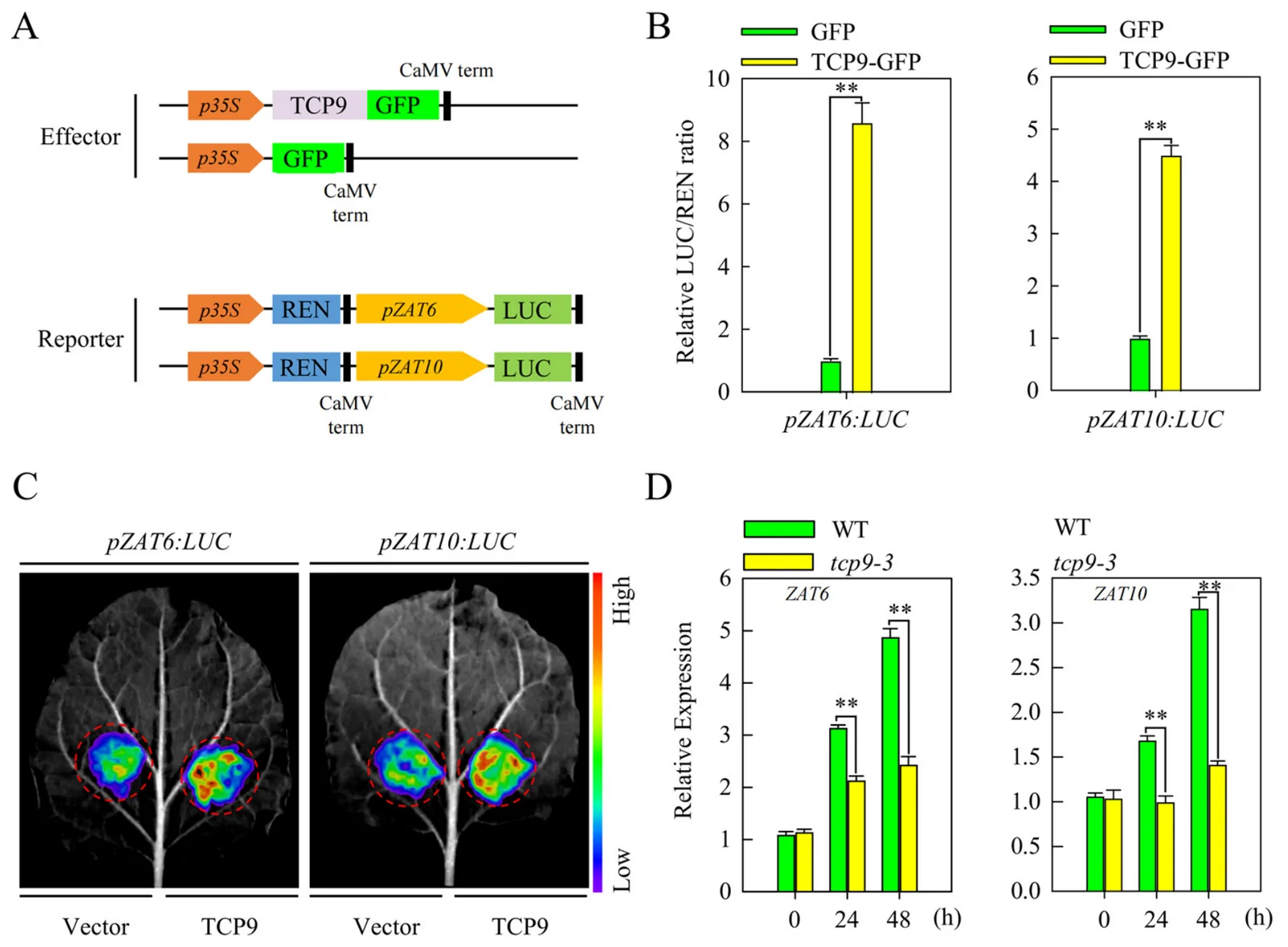
TCP9 enhances the promoter activity of TFs *ZAT6* and *ZAT10*. (**A**) A schematic shows the dual-luciferase constructs used in a mesophyll protoplast expression system. (**B**) The TCP9 protein enhances the transcriptional activity of Cd tolerance-related TFs *ZAT6* and *ZAT10* promoters. REN served as an internal control, and the LUC/REN ratio indicates the relative activity of these promoters. (**C**) The TCP9 protein activates the activity of *ZAT6* and *ZAT10* promoters in *N*. *benthamiana* leaves. *ZAT6p:LUC* and *ZAT10p:LUC* vectors were co-infiltrated with either an empty vector or a *TCP9* overexpression vector. Images were captured 48 h post-infiltration, with consistent results across at least three replicates. LUC luminescence signals in *Nicotiana benthamiana* are marked with red circles. (**D**) *ZAT6* and *ZAT10* expression levels in WT and *tcp9-3* mutant plants were measured at 0, 24, and 48 h after treatment with 30 μM CdSO_4_. Data are shown as mean ± SE from three replicates, with asterisks marking significant differences (Student’s *t*-test, ** *p* < 0.01).

TFs often form cascading amplification regulatory modules that play important roles in enabling plants to respond rapidly and precisely to biotic and abiotic stresses. To investigate the molecular mechanism by which TCP9 enhances Cd tolerance in *Arabidopsis*, we employed a dual-luciferase reporter assay in *Arabidopsis* mesophyll protoplasts to identify potential regulatory modules of TCP9. The results showed that, compared with the GFP control, the TCP9-GFP fusion protein significantly enhanced *LUC* expression driven by the *ZAT6* and *ZAT10* promoters (Figure 6A,B), but had no significant effect on *LUC* expression driven by the *ZAT12*, *FIT*, *bHLH38*, *bHLH39*, *bHLH100*, and *bHLH101* promoters (Figure S4A,B). Subsequent luciferase reporter assays in *Nicotiana benthamiana* leaves further validated that TCP9 significantly enhanced *LUC* expression driven by the *ZAT6* and *ZAT10* promoters, relative to the empty vector control (Figure 6C). Furthermore, RT-qPCR analysis indicated that the expression levels of *ZAT6* and *ZAT10* in the *tcp9-3* mutant were significantly lower than those in the WT at 24 and 48 h after Cd treatment (Figure 6D).

**Figure 7:**
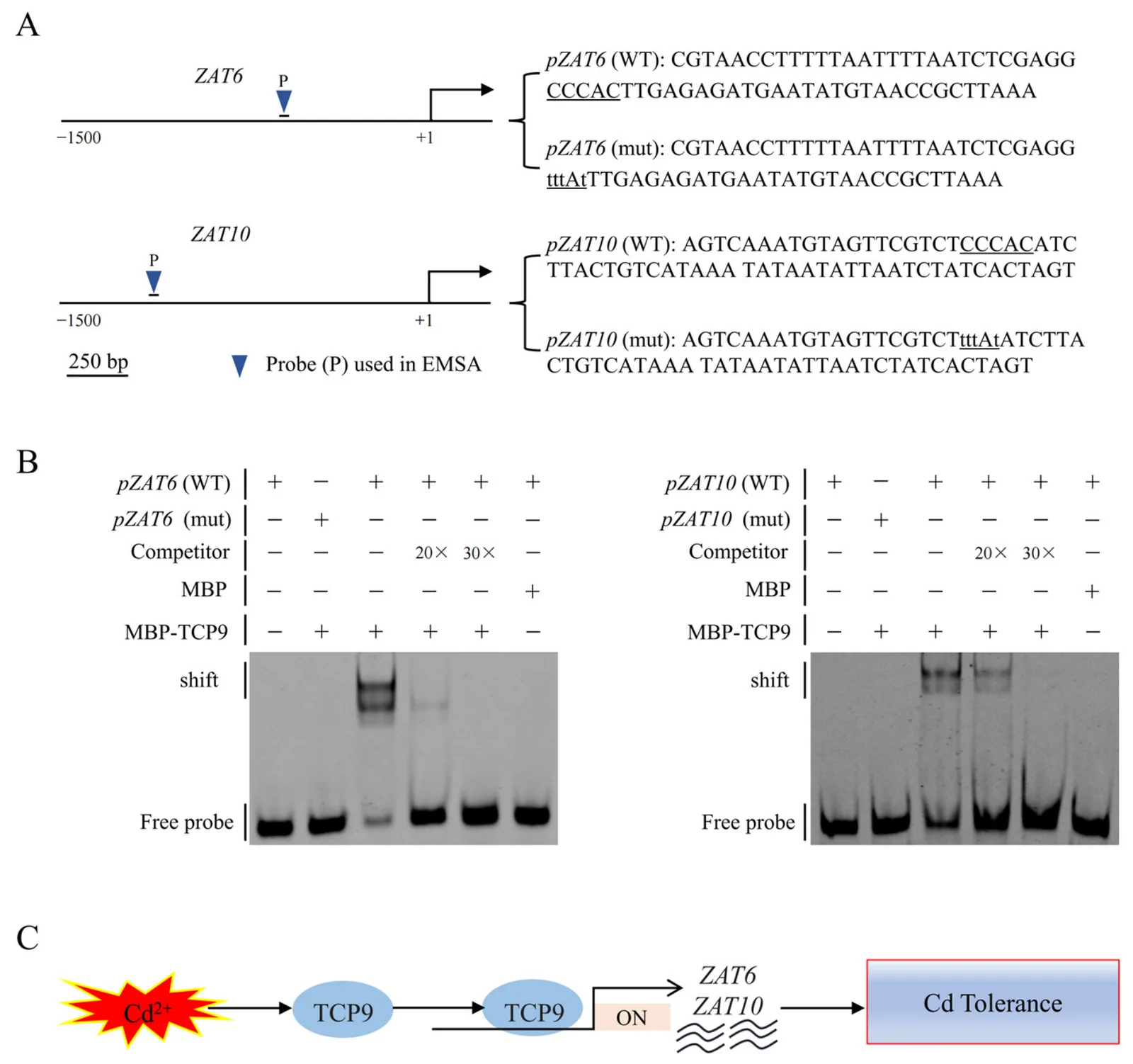
TCP9 directly activates *ZAT6* and *ZAT10* expression. (**A**) Schematic representations of the promoter sequence selection for *ZAT6* and *ZAT10* utilized in the EMSA are provided. Triangular markers denote the positions of the sequences used in the EMSA. (**B**) The EMSA confirmed the direct interaction of TCP9 with the promoters of *ZAT6* and *ZAT10* in vitro. (**C**) The proposed model shows that TCP9 facilitates Cd tolerance in *Arabidopsis* through regulating *ZAT6* and *ZAT10* expression. Arrows indicate promotion or gene activation.

Subsequently, we developed DNA probes specific to sequences containing the CCCAC motif within the promoters of *ZAT6* and *ZAT10*, which were labeled with *Cy5* fluorescence. Concurrently, the core CCCAC motif was altered to tttAt to produce mutant probes (Figure 7A). Electrophoretic mobility shift assay (EMSA) results indicated that both the negative control (DNA probe alone) and the MBP protein control showed only free unbound DNA probes at the bottom of the gel. In contrast, two distinct bands were detected in the MBP-TCP9 binding group, corresponding to the MBP-TCP9/DNA probe complex and unbound free DNA probe, respectively. Furthermore, the binding band of MBP-TCP9 with the DNA probe progressively diminished as the concentration of the unlabeled competitor probe increased. Importantly, the addition of the mutant probe, in which the core CCCAC motif was altered to tttAt, resulted in the absence of the MBP-TCP9/mutant probe complex, with only a single band corresponding to the free DNA probe observed on the gel (Figure 7B). Hence, TCP9 protein can directly bind to the CCCAC *cis*-acting elements in the promoters of *ZAT6* and *ZAT10* in vitro. Collectively, these results provide compelling evidence that TCP9 directly binds the *ZAT6* and *ZAT10* promoters to activate their transcription, thereby enhancing the tolerance of *Arabidopsis* to Cd toxicity.

## 3. Discussion

Soil contamination with Cd has become a critical global concern, significantly impacting crop production and posing risks to human health [3]. Elucidating the molecular mechanisms governing plant responses to Cd toxicity is essential for enhancing plant tolerance. Numerous studies have indicated that TFs are integral to plant responses to Cd toxicity, as they regulate ROS accumulation, antioxidant capacity, and Cd uptake and translocation [21]. Notably, *ZAT6* and *MYB4* enhance the tolerance of *Arabidopsis* to Cd toxicity by upregulating genes involved in the biosynthesis pathways of GSH and PC [14,23]. Moreover, our prior study has demonstrated that *ZAT10* enhances the tolerance of *Arabidopsis* to Cd toxicity by modulating ROS accumulation and the antioxidant system [16], although the upstream regulatory genes and mechanisms have yet to be elucidated. In the present study, we have identified that TCP9 enhances Cd tolerance by modulating the expression of *ZAT6* and *ZAT10* in *Arabidopsis*.

### 3.1. TCP9 Positively Regulates Cd Tolerance in Arabidopsis

The utilization of *Arabidopsis T-DNA* insertion mutants has been instrumental in elucidating gene functions and dissecting the genetic pathways associated with specific phenotypes [40,41]. Through the systematic screening of *T-DNA* insertion mutants, pivotal genes in the ethylene (ET), JA, and abscisic acid (ABA) signaling pathways have been identified [42,43]. Additionally, this approach has facilitated the discovery of regulatory genes that play crucial roles in plant responses to various abiotic and biotic stresses [44]. In this study, we conducted a phenotypic screening of a series of *T-DNA* insertion mutants under Cd toxicity conditions. Our results identified that the mutant SALK_035853 exhibited a significant reduction in primary root length and fresh weight compared to the WT (Figure S1 and Figure 2). This phenotype is attributed to a loss-of-function mutation caused by *T-DNA* insertion into the 5′ untranslated region (UTR) of the AT2G45680 gene, which encodes *TCP9* [34]. Further investigation revealed that Cd toxicity induces the transcription of *TCP9*. Additionally, the *tcp9* mutant line demonstrated increased Cd accumulation relative to the WT under Cd toxicity (Figure 3). These findings suggest that *TCP9* functions as a positive regulatory gene conferring enhanced Cd tolerance in *Arabidopsis*.

Extensive research has established that TFs are integral components of the regulatory network that governs plant tolerance to Cd toxicity. These TFs modulate the expression of genes associated with Cd detoxification and tolerance, thereby affecting plant adaptation to Cd toxicity. Notably, WRKY, ERF, MYB, bHLH, and bZIP families have been extensively characterized for their roles in mediating Cd tolerance, with their regulatory pathways well-documented across various model plants and crops [20]. However, the specific members of other TF families involved in Cd tolerance and their underlying regulatory mechanisms remain largely unknown. In this study, we present compelling evidence that TCP9 is implicated in Cd tolerance in *Arabidopsis*. Previous studies have identified that class I of the TCP family in *Arabidopsis* comprises 13 members (TCP6, TCP7, TCP8, TCP9, TCP11, TCP14, TCP15, TCP16, TCP19, TCP20, TCP21, TCP22, and TCP23), which play a crucial role in the plant’s response to abiotic and biotic stresses by regulating phytohormone biosynthesis and signaling pathways [30]. Crucially, TCP9 is implicated in immune defense against pathogens by modulating the expression of *Isochorismate Synthase 1* (*ICS1*) and negatively impacting JA biosynthesis through direct repression of the JA synthesis gene *LOX2* [30,34]. Furthermore, *TCP9* expression is significantly upregulated in response to nematode infection. Additionally, TCP9 is pivotal in maintaining ROS homeostasis, thereby influencing root plasticity and enhancing the plant’s capacity to manage nematode infections [39]. Therefore, our study uncovers a novel function for TCP9, a member of class I.

### 3.2. TCP9 Improves Plant Tolerance to Cd Toxicity by Reducing Oxidative Damage from Cd Exposure

Recent studies have consistently demonstrated that TFs modulate Cd tolerance through alterations in ROS levels and the capacity of the antioxidant system. For instance, in *Arabidopsis*, bZIP30 enhances Cd tolerance by activating *GST11* [45]. In contrast, WRKY12 diminishes *Arabidopsis* tolerance to Cd toxicity by directly repressing the expression of *GSH1* and genes involved in PC synthesis [25]. Conversely, ZAT6 enhances *Arabidopsis* tolerance to Cd by directly activating *GSH1* expression and synergistically regulating the expression of PC synthesis-related genes, including *GSH1*, *GSH2*, *PCS1*, and *PCS2* [14]. Similarly, ZAT10 increases *Arabidopsis* Cd tolerance by reducing ROS accumulation and boosting the activity of protective enzymes [16]. In maize, ZmWRKY4 and ZmWRKY64 contribute to improved Cd tolerance by modulating ROS homeostasis [46,47]. Furthermore, our previous study has identified a positive feedback loop between H_2_O_2_ and *CaWRKY41* expression, which collaboratively regulates the response of pepper to Cd toxicity [5]. In alignment with these findings, our study similarly demonstrates that, in comparison to the WT, the *tcp9* mutant shows increased H_2_O_2_ accumulation (Figure 4 and Figure 5A) and reduced contents of CAT, POD, and APX under Cd toxicity (Figure 5B–D). These results provide compelling evidence for a novel role of TCP9 in augmenting plant tolerance to Cd toxicity.

### 3.3. TCP9 Modulates Cd Tolerance Through the Direct Regulation of ZAT6 and ZAT10 Expression in Arabidopsis

Currently, while numerous TFs have been identified as playing significant roles in plant responses to Cd toxicity, the research on regulatory modules among TFs related to Cd tolerance is relatively underdeveloped compared to advancements in understanding plant responses to nitrogen starvation, phosphorus starvation, Fe starvation, cold stress, thermotolerance, and plant immunity [20,48,49]. Furthermore, the molecular mechanisms by which plants enhance Cd tolerance through the cascade amplification of TFs remain poorly understood. This study demonstrates that TCP9 can directly bind to the promoters of *ZAT6* and *ZAT10*, thereby activating their transcription and enhancing *Arabidopsis* tolerance to Cd toxicity. Initially, transient expression assays conducted with *Arabidopsis* mesophyll cell protoplasts demonstrated that the TCP9-GFP fusion protein significantly enhanced the promoter activities of *ZAT6* and *ZAT10*, relative to the control GFP protein (Figure 6B). In contrast, the TCP9-GFP fusion protein did not affect the promoter activities of *ZAT12*, *FIT*, *bHLH38*, *bHLH39*, *bHLH100*, and *bHLH101* (Figure S4). In addition, a significant reduction in *ZAT6* and *ZAT10* was detected in *tcp9* mutant lines under Cd toxicity compared to the WT (Figure 6D). Additionally, EMSAs confirmed the binding of the TCP9 protein to the promoters of *ZAT6* and *ZAT10* in vitro (Figure 7A,B). Collectively, these findings offer robust evidence that TCP9 directly activates the expression of *ZAT6* and *ZAT10*, thereby enhancing the tolerance of *Arabidopsis* to Cd toxicity.

Overall, this study advances the current knowledge of transcriptional regulation in plant Cd tolerance and offers a theoretical framework for identifying target genes. Future research should concentrate on two primary areas: (1) elucidating the molecular network of plant Cd responses, especially identifying TFs or signaling proteins that interact with TCP9; and (2) exploring TCP9-mediated cross-adaptation to abiotic and biotic stresses, with the goal of genetically enhancing multi-stress tolerance.

## 4. Materials and Methods

### 4.1. Plant Materials and Growth Conditions

*Arabidopsis thaliana* ecotype Columbia-0 (Col-0) was utilized as the wild-type control. The *TCP9 T-DNA* insertion line (*tcp9*, SALK_035853) [34], obtained from the Arabidopsis Biological Resource Center, was identified using the left primer (*LP*), right primer (*RP*), and the *T-DNA* left-border primer *LBb1.3*. For the Cd treatment assay, seeds were surface sterilized, vernalized at 4 °C for 48 h, and subsequently sown in 13 × 13 cm square petri dishes containing half-strength Murashige and Skoog (MS) [50] agar medium (Sigma, catalog no. A1296, Darmstadt, Germany) with varying concentrations of CdSO_4_ (0, 30, 60, and 90 μM). Root length and fresh weight were measured, and photographs were taken after 9 d. To assess the levels of Cd and H_2_O_2_, as well as the CAT, APX, and POD, 7-day-old *tcp9* and wild-type seedlings were transferred to half-strength MS medium supplemented with 30 μM CdSO_4_ for 3 and 5 d. Subsequently, the samples were harvested and analyzed. For analyzing gene expression under Cd stress, 7-day-old seedlings were transferred to a half-strength MS agar medium, which was supplemented with specified concentrations of CdSO_4_ for a predetermined duration. The seedlings were collected for RT-qPCR analysis. *Arabidopsis* seedlings were cultivated in an incubator set to 22 ± 2 °C and 70 ± 5% relative humidity with a 16-h light/8-h dark photoperiod and a photosynthetic photon flux density (PPFD) of 100 µmol photons m^−2^ s^−1^. In parallel, *N*. *benthamiana* plants were grown at 25 ± 2 °C under a 16-h light/8-h dark cycle for transient expression assays.

### 4.2. Molecular Cloning and Plasmid Construction

To create complementary lines of *tcp9* mutants, the genomic DNA fragment of *TCP9* with its promoter was cloned into the *pCAMBIA1300* vector by *SacI* and *SalI* sites, and *Arabidopsis* was transformed using the floral dip method. For transient expression assays, effector vectors were made by cloning *TCP9* coding sequences from *Arabidopsis* cDNA into the *p19-GFP* vector by the *XbaI-SpeI* sites, producing the effector construct *pCaMV35S-TCP9-GFP*. Reporter vectors were constructed by cloning promoter fragments of *ZAT6*, *ZAT10*, and *ZAT12* into the *pGreen-0800-LUC* vector by the *Hind*III*-Bam*HI sites, producing the reporter constructs *pZAT6:LUC*, *pZAT10:LUC*, and *pZAT12:LUC*. For EMSA, the CDS fragment of *TCP9* was cloned into the *pMAL-c4X* vector (NEB, http://www.neb-china.com/) by the *EcoRI-XbaI* sites. The primers used for the generation of these vectors are detailed in Table S1.

### 4.3. Transient Expression Assays

Transient expression assays were conducted using *Arabidopsis* mesophyll protoplasts as described previously [51]. In brief, the plasmids were extracted and purified from *Escherichia coli* DH5α cultures using the Endofree Maxi Plasmid Kit (DP117, TIANGEN, Beijing, China). Effector (such as *pCaMV35S-TCP9-GFP* and *pCaMV35S-GFP*) and reporter (such as *pZAT6:LUC*, *pZAT10:LUC*, and *pZAT12:LUC*) plasmids were transformed into *E. coli* DH5α, plated on LB agar, and incubated overnight. Colonies were then grown in 200 mL of Terrific Broth (TB) medium with 75 μg mL^−1^ ampicillin or 50 μg mL^−1^ kanamycin at 37 °C with shaking. Plasmids were extracted, precipitated with isopropanol and 5 M NaCl, washed with 70% ethanol, and dissolved in ultrapure distilled water (Thermo Fisher Scientific Product No.: 10977035, Waltham, MA, USA). The final plasmid concentration was set to 2000 ng µL^−1^. The 200 μL protoplasts were co-transfected with 20 μg effector and 20 μg reporter plasmids and then incubated in WI buffer for 10 h. Subsequently, a dual-luciferase reporter assay was performed. Briefly, protoplast cells were lysed and the supernatant was collected by centrifugation at 10,000× *g* for 5 min. Firefly and renilla luciferase activities were measured using a dual-luciferase assay kit (Yeasen, Cat# 11402ES60, Shanghai, China), following the manufacturer’s instructions. Relative promoter activity was calculated as the ratio of firefly luciferase activity to renilla luciferase activity.

Transient transcription assays were conducted on *N*. *benthamiana* plants. In brief, the effector and reporter constructs were transformed into *Agrobacterium* GV3101 (*pSoup*). Then, leaves of 6- to 8-week-old plants were infiltrated with the *Agrobacterium* suspension (OD_600_ = 0.5) and incubated for 48 h at 28 ± 2 °C. Firefly luciferase luminescence was photographed and quantified after applying 1 mM luciferin (Thermo Fisher Scientific, Product No.: 88294, Pierce, Waltham, MA, USA) using a Night SHADE LB985 system (Berthold, Germany).

### 4.4. DAB Staining

DAB staining was conducted following established protocols [16]. Briefly, seedlings were meticulously immersed in a DAB solution (1 mg mL^−1^, pH 3.8) and incubated at 25 °C for 12 h. Following the staining process, the seedlings were subjected to thorough bleaching with 70% ethanol (*v*/*v*). Subsequently, image capture was performed using a Stereo Microscope from Leica (Wetzlar, Germany).

### 4.5. Measurement of Cd Content

The determination of Cd content was conducted following a previous study [16]. Briefly, root and shoot samples from *Arabidopsis* seedlings were rinsed three times with ultrapure water, with each rinse lasting 3 min. After weighing, the samples underwent digestion using 0.5 mL of concentrated HNO_3_ for roots and 1 mL for shoots. Once the solution was clarified, it was diluted to a total volume of 10 mL and thoroughly mixed. Subsequently, the solution was filtered and the Cd concentration was measured using inductively coupled plasma-atomic emission spectrometry (Thermo Scientific, USA).

### 4.6. Measurement of H_2_O_2_ and Enzyme Levels

Approximately 80 mg of the sample was subjected to cryogenic grinding using a tissue homogenizer. The resultant pulverized samples were then homogenized in a phosphate buffer (600 μL, 50 mM, pH 7.0) and subsequently centrifuged at 3000× *g* for 10 min at 4 °C. Following this preparation, the contents of H_2_O_2_, POD, CAT, and APX were evaluated using an enzyme-linked immunosorbent assay (ELISA) kit (Shanghai Bangyi Biotechnology Co., Ltd., China, catalog no. BYE97057 for H_2_O_2_, catalog no. BYE97047 for POD, catalog no. BYE97048 for CAT, and catalog no. BYE97079 for APX). Then, microtiter plate wells were coated with purified H_2_O_2_, POD, CAT, and APX antibodies to create a solid-phase antibody. Samples and horseradish peroxidase-labeled antibodies were added, forming an antibody–antigen-enzyme complex. After washing, substrate solution was added, and absorbance at 450 nm was measured using a Tecan Infinite 200 Pro Plate Reader (Tecan Group Ltd., Männedorf, Switzerland). Subsequently, standard curves were generated using the standard samples supplied with the ELISA kit. The concentrations of H_2_O_2_, POD, CAT, and APX were quantified from their corresponding absorbance values, with enzyme activity expressed as units mg^−1^ FW.

### 4.7. EMSA

EMSA was conducted by transforming either recombinant plasmids or empty vectors into *E. coli* BL21. Induction of the MBP-TCP9 fusion protein was achieved using 0.4 mM isopropyl β-D-1-thiogalactopyranoside (IPTG), followed by incubation at 16 °C for 16 to 20 h in LB medium. Protein purification was performed utilizing amylose resin (New England Biolabs, Beijing, China), with subsequent washing in CB buffer (20 mM Tris-HCl, 100 mM NaCl, 10 mM EDTA, pH 7.4) and elution in a buffer comprising 20 mM Tris-HCl, 100 mM NaCl, 10 mM EDTA, and 0.5 mM maltose, at pH 7.4. The expression of recombinant TCP9 protein was verified by SDS-PAGE analysis. Then, MBP-TCP9 protein was incubated with probes in EMSA binding buffer (Beyotime Biotechnology, Shanghai, China) at 25 °C for 30 min. *Cy5*-labeled probes were synthesized from *ZAT6* and *ZAT10* promoter fragments, as detailed in Table S1. Samples were resolved on 12% native polyacrylamide gels, and the gel was visualized by a LI-COR Odyssey Infrared Imaging System to detect the fluorescent label LI-COR Biosciences, Lincoln, NE, USA).

### 4.8. Expression Analysis

Briefly, RNA was extracted from *Arabidopsis* seedlings using the RNAprep Pure Plant Kit (Tiangen Biotech, Beijing, China). Then, 500 ng of RNA was used to synthesize cDNA with the HiScript III RT SuperMix for qPCR (Cat# R323-01, Nanjing Vazyme Biotech Co., Ltd., Nanjing, China). Gene expression was quantified on a Light-Cycler 480 Real-Time PCR System (Roche, Switzerland) with ChamQ SYBR qPCR Master Mix (Cat# Q711-02, Nanjing Vazyme Biotech Co., Ltd., Nanjing, China), using specific primers listed in Table S1, and normalized to *UBQ10*.

### 4.9. Statistical Analysis

Statistical differences among groups were analyzed using Duncan’s multiple range test, with distinct letters indicating significance (lowercase for *p* < 0.05, uppercase for *p* < 0.01). Student’s *t*-test assessed differences between groups, marked by asterisks (* for *p* < 0.05, ** for *p* < 0.01). Analyses were conducted using Data Processing System (DPS, 9.5) software. Each experiment had at least three independent biological replicates. Results were shown as means ± standard error (SE).

### 4.10. Accession Numbers

The sequence data for this study can be found on the Arabidopsis Information Resource (TAIR, https://www.arabidopsis.org/) with these identifiers: *TCP9* (AT2G45680), *ZAT6* (AT5G04340), *ZAT10* (AT1G27730), *ZAT12* (AT5G59820), *FIT* (AT2G28160), *bHLH38* (AT3G56970), *bHLH39* (AT3G56980), *bHLH100* (AT2G41240), *bHLH101* (AT5G04150), and *UBQ10* (AT4G05320).

## 5. Conclusions

Based on our findings, we propose a streamlined working model to elucidate the role of TCP9 in enhancing the tolerance of *Arabidopsis* to Cd toxicity (Figure 7C). Cd toxicity triggers the expression of *TCP9*, which subsequently binds directly to the promoters of the zinc-finger TFs *ZAT6* and *ZAT10*, thereby activating their transcription and enhancing the plant’s tolerance to Cd toxicity. Additionally, identifying interacting regulatory partners of TCP9 and its downstream target genes is crucial for fully understanding the molecular mechanisms of Cd tolerance. Overall, our findings provide new insights into the molecular function of TCP9 in plant responses to Cd toxicity.

## Data Availability

The data utilized in this study are provided within the article and its accompanying Supplementary Materials. Additionally, the data that underpin the findings of this study are available from the corresponding author upon request.

## Supplementary Materials

The following supporting information can be downloaded at: https://www.mdpi.com/article/10.3390/plants15172563/s1.
