# Supplementary material for: Transcription Factor TCP9 Enhances Arabidopsis Tolerance to Cadmium Toxicity via ZAT6 and ZAT10 Activation
Source: Plants (Basel). 2026 Aug 24;15(17):2563. doi: 10.3390/plants15172563 (PMC13567022; doi:10.3390/plants15172563)
Supplement: Supplementary file 1 [file plants-15-02563-s001.zip › plants-4429111-supplementary.pdf]

Supplementary Data

Supplementary Figures

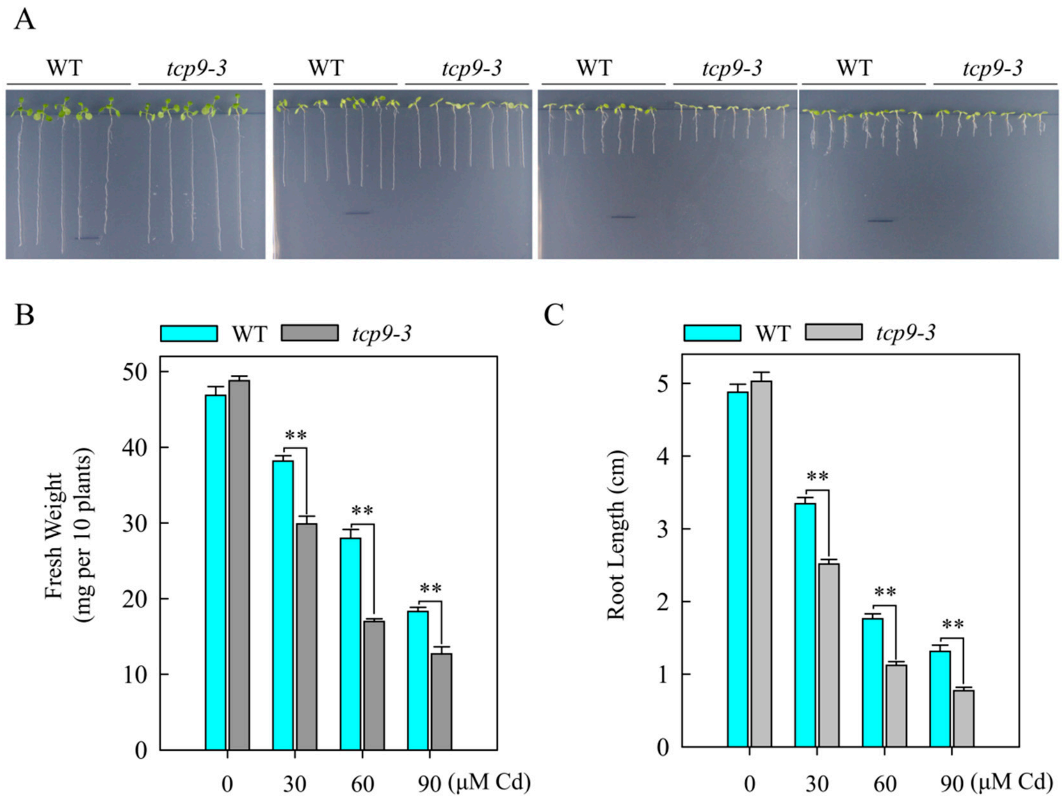

Supplementary Figure S1. The *tcp9-3* mutant of transcription factor *TCP9* is sensitive to Cd toxicity.

(A) A phenotypic comparison was conducted between WT and *tcp9-3* mutant seedlings under conditions of Cd toxicity. Seven-day-old seedlings were cultivated on 1/2 MS medium, both in the absence and presence of $CdSO_4$ at concentrations of 30, 60, and 90 μM. A white scale bar represents 1 cm. The experiment was replicated independently three times, yielding consistent results.

(B and C) Measurements of root length **(B)** and fresh weight **(C)** were taken from seven-day-old WT and *tcp9-3* seedlings grown vertically on 1/2 MS agar plates with varying concentrations of $CdSO_4$. Data are expressed as the mean ± standard error (SE) with a sample size of n = 3. Asterisks denote statistically significant differences as determined by Student's t-test (** $P<0.01$), while NS indicates no significant difference.

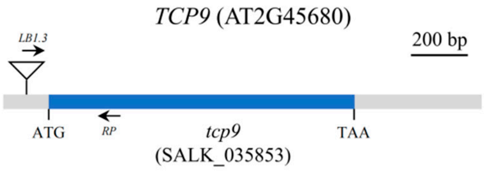

Supplementary Figure S2. A schematic representation of the *T-DNA* insertion mutant *tcp9-3* of the *TCP9* gene.

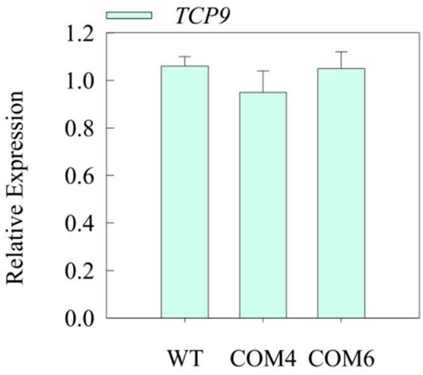

Supplementary Figure S3. *TCP9* transcript levels were assessed via RT-qPCR in WT and *TCP9pro:TCP9* complementation lines COM4 and COM6.

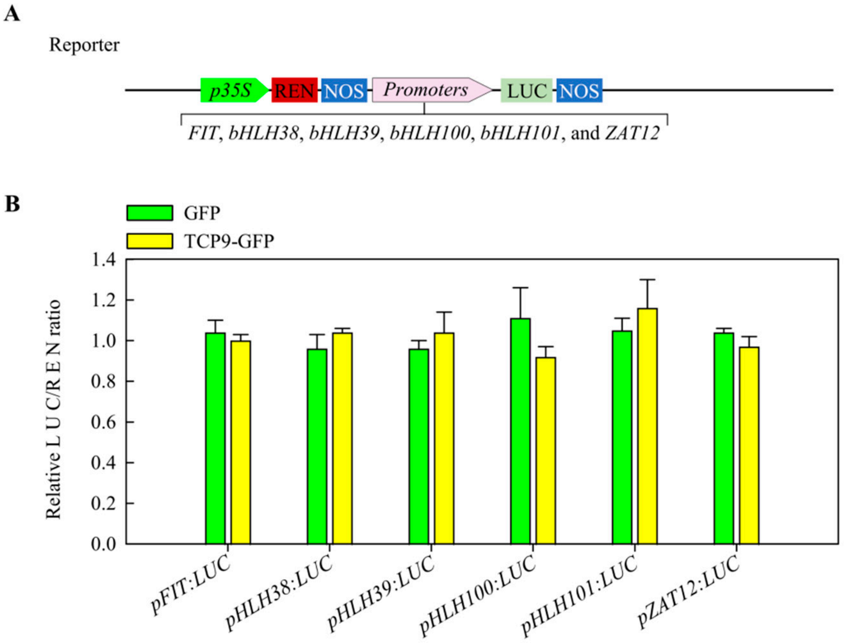

Supplementary Figure S4. TCP9 does not affect the transcriptional activity of other transcription factor promoters.

(A) A schematic illustration of the dual-luciferase reporter constructs utilized in the mesophyll protoplast transient expression system.

(B) TCP9 does not impact the promoter transcriptional activity of the TFs *FIT*, *bHLH38*, *bHLH39*, *bHLH100*, *bHLH101*, and *ZAT12* genes.

Supplementary Table

Supplementary Table S1 The primers used in this study

Primer Name	Forward primer (5'-3')	Reverse primer(5'-3')
**Primers used for DNA constructs**		
TCP9 (*CDS*)*-Zero*	ggatcttccagagatATGGCGACAATTCAGAAGCTTG	ctgccgttcgacgatTCAGTGGTTCGATGACCGTGC
p1300-TCP9	catgattacgaattcgagctcGGTTAAGCGTTTTACTTATG	cttgcatgcctgcaggtcgacTTTCATTTTAGATAGAAATA
**Primers used for *tcp9-3* mutant identification**		
LB1.3	ATTTTGCCGATTTCGGAAC	
tcp9-3-LP	TGTGACCGGTTTAATCAAAGG	
tcp9-3-RP	TTCCTTCAACCTTCGTGTGAC	
**Primers used for transient expression assays**		
pCaMV35S-TCP9-GFP	gacaagcttggtacctctagaATGGCGACAATTCAGAAGCT	gcccttgctcaccatactagtGTGGTTCGATGACCGTGCTGTT
pFIT:LUC	gtcgacggtatcgataagcttAGAAACATTACAGACCGAAATTT	cgctctagaactagtggatccTTGTGTGTTTTTGTCAATGAAGAA
pbHLH38:LUC	gtcgacggtatcgataagcttTTTATAGTTATGTTATGTCGTCCTT	cgctctagaactagtggatccTTTTTGCTTAATCAAGGACAAGG
pbHLH39:LUC	gtcgacggtatcgataagcttTTCGAAGAAGCAACCTTCTCTGT	cgctctagaactagtggatccTTTTGCTTACTAAGGACAAGTATT
pbHLH100:LUC	gtcgacggtatcgataagcttCTACGTTCTAGACGTTCAGTAAAG	cgctctagaactagtggatccTTTGAGTTTTAGATAGTTACTCTGTT
pbHLH101:LUC	gtcgacggtatcgataagcttCTCACTAGCTAATTCGTGGATATTT	cgctctagaactagtggatccTTTTTTTGTCCACTTATGTTATGTGT
pZAT6:LUC	gtcgacggtatcgataagcttTAATCCATGATTTAGAACA	cgctctagaactagtggatccTATCTTGAAGACTAGCTAC
pZAT10:LUC	gtcgacggtatcgataagcttTTTATCATGTATTACAAGTT	cgctctagaactagtggatccTAAGTTAAAGATTCTGAGG
pZAT12:LUC	gtcgacggtatcgataagcttGGAAGTGTGTAAGTATAATA	cgctctagaactagtggatccTTTTCTTCTGATGATGATGA
**Primers used for EMSA**		
ZAT6-Cy5-EMSA (WT)	TTTAAGCGGTTACATATTCATCTCTCAA*GTGGG*CCTCGAGATTAAAATTAAAAAGGTTACG	CGTAACCTTTTTAATTTTAATCTCGAGG*CCCAC*TTGAGAGATGAATATGTAACCGCTTAAA
ZAT6-Cy5-EMSA (mut)	TTTAAGCGGTTACATATTCATCTCTCAA*aTaaa*CCTCGAGATTAAAATTAAAAAGGTTACG	CGTAACCTTTTTAATTTTAATCTCGAGG*ttAt*TTGAGAGATGAATATGTAACCGCTTAAA

ZAT10-Cy5-EMSA (WT)	AGTCAAATGTAGTTCGTCT*CCCAC*ATCTTACTGTCATAAATATAATATTAATCTATCACTAGT	ACTAGTGATAGATTAATATTATATTTATGACAGTAAGAT*GTGGG*AGACGAACTACATTTGACT
ZAT10-Cy5-EMSA (mut)	AGTCAAATGTAGTTCGTCT*tttAt*ATCTTACTGTCATAAATATAATATTAATCTATCACTAGT	ACTAGTGATAGATTAATATTATATTTATGACAGTAAGAT*aTaaa*AGACGAACTACATTTGACT
**Primers used for RT-qPCR**		
qUBQ10	TCCTCAGGCTCCGTGGTGGT	AGCCTCTGCTGGTCCGGAGG
qTCP9	CCAAAAAGAGAGTGGAGGGAG	TCAAGACAAAGTGGTGAGCCC
qZAT6	TGACCTGCCTTCTTCTTCGT	GCCTTGTCACAGACGCTACA
qZAT10	GTCAACGGAGACGACGAAGTCA	AGTTGTAGTGGTGTCACTTTATGCT
qZAT12	GAGTCACAAGAAGCCTAACAACGA	AAGCCACTCTCTTCCCACTGCTA
